# Homologous recombination in human embryonic stem cells using CRISPR/Cas9 nickase and a long DNA donor template

**DOI:** 10.1007/s13238-014-0032-5

**Published:** 2014-03-14

**Authors:** Zhili Rong, Shengyun Zhu, Yang Xu, Xuemei Fu

**Affiliations:** 1 Shenzhen Children’s Hospital, 7019 Yitian Road, Shenzhen, 518026 China; 2 Division of Biological Sciences, University of California, San Diego, 9500 Gilman Drive, La Jolla, CA 92093 USA; 3 Chongqing Medical University, Chongqing, 400016 China

**Dear Editor,**

Genome editing of human embryonic stem cells (hESCs) is critical for basic biological research and regenerative medicine. However, until a few years ago, gene targeting technologies to disrupt, repair or overexpress genes in hESCs had been very inefficient and thus could not be routinely used. Recent technical breakthrough includes bacterial artificial chromosome based high efficiency gene targeting (Song et al., 2010), as well as the successful engineering of two systems of site-specific nucleases, zinc-finger nucleases (ZFNs) and transcription activator-like effector nucleases (TALENs) (Hockemeyer et al., 2009; Hockemeyer et al., 2011). The two engineered nucleases are composed of programmable and sequence-specific DNA-binding modules, which bring the nucleases to specific genomic site to introduce a DNA double-strand break. However, these two technologies have several limitations, including the time-consuming and labor-intensive experimental design, and the risk of off-targeting mutations (Gaj et al., 2013). More recently, a new genome-editing technology, denoted the clustered regularly interspaced short palindromic repeats (CRISPR)/CRISPR-associated (Cas) system, has been developed for efficient gene targeting in cells of various species, including zebrafish (Hwang et al., 2013), mouse (Wang et al., 2013), monkey (Niu et al., 2014), and human (Cong et al., 2013; Mali et al., 2013b). In this system, Cas9 nuclease is targeted to a specific genomic site by complexing with a guide RNA, which hybridizes a 20-nucleotide DNA sequence immediately preceding an NGG motif, introducing a double-strand break three nucleotides upstream of the NGG motif (Jinek et al., 2012). Compared to ZFNs and TALENs, CRISPR/Cas9 system offers simple experimental design and very high targeting efficiency (Ding et al., 2013). However, some studies have also raised the concern about the off-target mutation effect of this system (Hsu et al., 2013; Mali et al., 2013a). To reduce the off-target mutations, a D10A mutant nickase version of Cas9 (Cas9n) has been developed to replace wild type Cas9 and shown to increase the ratio of homology-directed repair (HDR) to nonhomologous end joining (NHEJ) (Cong et al., 2013; Mali et al., 2013b). However, the targeting efficiency of Cas9n is much lower than wild type Cas9, raising the concern that it cannot be applied in homologous recombination (HR) in hESCs.

To test whether Cas9n can induce precise gene targeting via homologous recombination at a gene locus not expressed in hESCs, we designed the experiment to employ Cas9n and a long DNA donor template to introduce a drug resistance gene into a lineage-specific locus. We picked the gene locus of pulmonary surfactant protein C (*SFTPC*), which is specifically expressed in pulmonary alveolar type II cells (Degryse et al., 1996). In the long DNA donor template, an IRES-Puro-pA expression cassette was inserted between the stop codon and the polyA signal sequence of *SFTPC* gene with two homologous arms around 1 kb. sgRNA-1 (#1 in Fig. 1A), which targeted the site about 170 bps upstream of the junction site of the two homologous arms, was designed according to the UCSC genome browser tracks developed by Zhang lab at MIT. While, sgRNA-2 (#2 in Fig. 1A), which targeted the junction site, was designed according to (N)_20_NGG rule. The Cas9n expression cassette, sgRNAs and donor DNA were electroporated into HUES3 hESCs. After puromycin selection, 37 and 36 clones survived with the transfection of sgRNA-1 and sgRNA-2, respectively. Five clones derived from sgRNA-1 transfection were found to be homologous recombinants at the upstream arm by PCR screening, while no clone derived from sgRNA-2 transfection was homologous recombinant (Fig. 1F). To confirm that the clones with homologous recombination at 5′ homologous arm do not harbor random integration, the genomic DNA was examined by Southern blotting with the upstream, downstream and internal probes, and with two restriction enzymes. As shown in Fig. 1D, two clones were identified to have precise HR at both arms and no random integration. The other three clones were identified to have single-crossover at the upstream homologous arm (data not shown). By transiently expressing Flp, the F5 flanked selection cassette was removed, leading to the knock-in allele (Fig. 1E).

**Figure 1:**
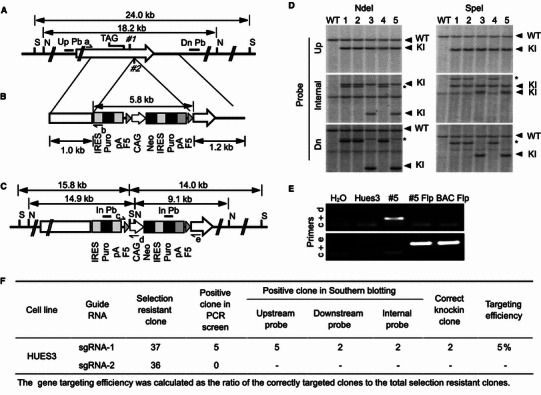
**CRISPR/Cas9 nickase induces precise gene targeting through homologous recombination in hESCs using a long DNA donor template**. (A) The endogenous human *SFTPC* locus. The arrow indicates *SFTPC* gene. The stop codon (TAG) and the two targeting sites (#1 and #2) of guide RNA are indicated. The locations of upstream and downstream probes for Southern blotting and the sizes of WT NdeI (N) and SpeI (S) restriction fragments are shown. (B) The long DNA donor template. The sizes and locations of homologous arms are indicated. The IRES-Puro-pA fragment and the F5 flanked selection cassette were inserted between the stop codon and the polyA signal sequence of *SFTPC* gene. Primers (a and b) used to screen homologous recombination at upstream arm are indicated. (C) The configuration of the knock-in allele. The location of internal probe for Southern blotting and the sizes of mutant NdeI and SpeI restriction fragments are indicated. Primers (c, d and e) used to screen clones after Flp transient expression are shown. (D) Southern blotting analysis of the clones with HR at upstream arm identified by PCR screening. Genomic DNA was digested with NdeI or SpeI and hybridized to the upstream, internal and downstream probes sequentially with stripping between probes. The WT and KI bands are indicated. Bands generated by single-crossover at the upstream homologous arm are indicated by asterisks. (E) Excision of selection cassette was confirmed by PCR analysis. Flp expression plasmid was introduced into the HUES3 KI cells by electroporation. Genomic DNA isolated from individual clones was examined by PCR. Primers c and d amplify a 264-bp fragment in KI cells with the selection cassette, while primers c and e amplify a 329-bp fragment in KI cells after the selection cassette is excised. BAC plasmid without selection cassette was used as a positive control. (F) Summary of the frequency of homologous recombination in hESCs

In this study, we demonstrate that Cas9n can induce precise gene targeting via HR in hESCs with a long DNA donor template. The targeting efficiency is around 5% (2/37) in HUES3 cells (Fig.1F). In contrast to the oligonucleotide-mediated gene targeting with wild type Cas9 that enables homozygous targeting of both alleles, no homozygous knock-in clones were identified in this study. To achieve homozygous knock-in clones with long DNA templates, a “double nicking” strategy might be employed with paired offset guide RNAs to significantly increase the homozygous targeting efficiency (Ran et al., 2013). We found three single-crossover clones with homologous recombination occurred only at the 5′ homologous arm. Therefore, our findings highlight the importance to confirm the homologous recombination events at both arms of the introduced donor DNA. In this context, in order to identify correctly targeted clones, both the upstream and downstream HR events as well as random integration of the template should be verified.

In summary, our studies indicate that Cas9n can achieve precise gene targeting via HR in hESCs with a long DNA template, allowing high fidelity and complex genetic manipulation of hESCs. One such application is to develop lineage-specific reporter lines to trace the lineage differentiation of hESCs.

## Electronic supplementary material

Below is the link to the electronic supplementary material.

Supplementary material 1 (PDF 172 kb)
