# Supplementary material for: Homologous recombination in human embryonic stem cells using CRISPR/Cas9 nickase and a long DNA donor template
Source: Protein Cell. 2014 Mar 14;5(4):258–60. doi: 10.1007/s13238-014-0032-5 (PMC3978163; doi:10.1007/s13238-014-0032-5)
Supplement: Supplementary file 1 — Supplementary material 1 (PDF 172 kb) [file 13238_2014_32_MOESM1_ESM.pdf]

Supplementary Methods

CRISPR/Cas9 plasmids

The human codon-optimized Cas9D10A expression plasmid and sgRNA plasmid were obtained from Addgene (44720 & 41824). sgRNA-1 and sgRNA-2 were constructed using Gibson assembly according to the gRNA synthesis protocol provided by Church lab at Harvard. The targeting sites of sgRNA-1 and sgRNA-2 are 5'- gaggagcgctcgcgctgaccAGG-3' and 5'- tgcaaaagaccctttgcccgGGG-3' (The underlined capital bases indicate the protospacer-adjacent motif, PAM). DNA oligos used to construct sgRNA-1 and sgRNA-2 are as follows: sgRNA-1F, 5'-tttcttggctttatatatcttgtggaaaggacgaaacaccgaggagcgctcgcgctgacc-3'; sgRNA-1R, 5'-gactagccttattttaacttgctatttctagctctaaaacggtcagcgcgagcgctcctc-3'; sgRNA-2F, 5'-tttcttggctttatatatcttgtggaaaggacgaaacaccggcaaaagaccctttgcccg-3'; sgRNA-2R, 5'-gactagccttattttaacttgctatttctagctctaaaaccgggcaaagggtcttttgcc-3'.

Cell culture

The hESC line, HUES3, was cultured on mouse embryonic fibroblast feeder layer in Knockout DMEM supplemented with 10% KOSR, 10% plasmanate, 0.1 mM nonessential amino acids, 2 mM Glutamax, 1% penicillin/streptomycin, 10 ng/ml bFGF, and 55 μM

β-mercaptoethanol. Media were changed every day. Cells were passaged on feeder with TrypLE every 4-5 days.

Gene targeting in hESCs

For electroporation, around 4 million hESCs were trypsinized to single cells with TrypLE, washed once with PBS, and resuspended in 500 ul PBS. 10 ug pCAG-Cas9n plasmid, 4 ug sgRNA plasmid and 6 ug DNA donor template were mixed with the cells and then electroporated in a 4-mm cuvette. Electroporation parameters were 320V, 200 uF, and infinite resistance. Cells were then plated onto puromycin-resistant feeders with 2.5 uM ROCK inhibitor Y-27632. Two days later, purimycin was added into the media for 3 days. About 2 weeks after electroporation, surviving clones were picked and expanded for further characterization.
